# Bilateral Simultaneous Femoral Neck Fracture Mimicking Abdominal Pain in a Cerebral Palsy Patient

**DOI:** 10.1155/2014/925201

**Published:** 2014-11-23

**Authors:** P. Mariani, M. Buttaro, F. Comba, E. Zanotti, P. Ali, F. Piccaluga

**Affiliations:** Institute of Orthopaedics “Carlos E. Ottolenghi”, Italian Hospital of Buenos Aires, Potosí 4247, C1199ACK Buenos Aires, Argentina

## Abstract

Simultaneous bilateral femoral neck fractures are unusual lesions, generally associated with an underlying condition which causes impaired bone mineralization, triggered by an increased bone stress. We present a 24-year-old cerebral palsy patient, who was previously evaluated in another institution due to inability to walk, interpreted as abdominal pain. No alteration in blood analysis or abdominal X-rays was found. As no response to treatment was observed, a new abdominal X-ray was taken, which incidentally depicted bilateral medial femoral neck fracture. He was referred to our practice after a resection arthroplasty was offered in another institution. After admission, bilateral one-stage THA was performed. Several reports emphasize bone disease as a major precipitating factor, and there is an increased incidence of hip fractures in chronic epilepsy, renal osteodystrophy, and chronic steroid use. Femoral head resection has been proven to be effective in immobilized patients, whereas this was not a reasonable option in this patient who presented walking ability. Despite the treatment election, primary care physicians should be aware of and alert to the possibility of fractures in patients with neurological disorders and calcium metabolism alterations. Late diagnosis of orthopedic injuries in this type of patients may lead to permanent disability.

## 1. Introduction

Simultaneous bilateral femoral neck fractures are somewhat unusual lesions, generally associated with an underlying condition which causes impaired bone mineralization, triggered by an increased bone stress, for example, a violent muscle contraction [1, 2]. Although there is a paucity of reports of this type of injury, they are mostly associated with bone metabolism disorders like chronic steroid use, renal osteodystrophy, and hypocalcaemia. It has also been linked with transient osteopenia during pregnancy [1–6].

Severe cerebral palsy affects the capacity of communication and therefore diminishes the physician ability to elucidate any clinical situation.

We present a 24-year-old cerebral palsy patient, with a bilateral femoral neck fracture mimicking an acute abdominal pain.

## 2. Case Report

P. J., a 24-year-old domiciliary ambulatory patient, with a spastic diplegia from a cerebral palsy, and an associated severe cognitive deficit, sought attention at our institution after four months of inability to walk. He was formerly classified by the Gross Motor Function Classification System as a level II [7], since the patient had limitations and needed assistance from her parents when walking outdoor, however did not need to use any orthotic devices. His parents referred no trauma such as a fall or impact.

He was previously evaluated in another institution due to inability to walk and fetal position which was interpreted as abdominal pain. However no alteration was found in blood analysis or abdominal X-rays. Oral antispasmodics have been previously administered throughout a 2-month period with little response. As no improvement was observed, new blood analysis and a new abdominal X-ray were taken, which incidentally included one of the hips and depicted medial femoral neck fracture. The patient was then transferred to the orthopaedic centre of that institution for subsequent management, and a Girdlestone procedure was offered due to the patient's previous condition. As the parents did not accept the limitations inherent, he was referred to our hospital.

On physical examination, there was severe pain in both hips. Both lower extremities were in external rotation and both knees were flexed at 30 degrees, which according to the parents was not present before this episode. Laboratory findings revealed patient haemoglobin concentration of 7.9 g/dL (normal range, 14–18 g/dL), blood urea nitrogen (BUN) level of 19 mg/dL (normal range, 7–20 mg/dL), creatinine level of 0.8 mg/dL (normal range, 0.6–1.3 mg/dL), serum albumin level of 4.3 g/dL (normal range, 3.4–5.0 g/dL), and serum calcium of 6.5 mg/dL (normal range, 8.4–10.2 mg/dL). Detailed investigations showed a parathormone (PTH) level of 51 pg/mL (normal value < 65), beta-crosslaps (CTX) of 0.678, vitamin D of 19.5 ng/mL (normal value > 30), and FSH of 19 UI/mL. Serum levels of sodium and potassium were normal. Computed tomography (CT) scanning of the brain revealed no focal brain lesion.

**Figure 1:**
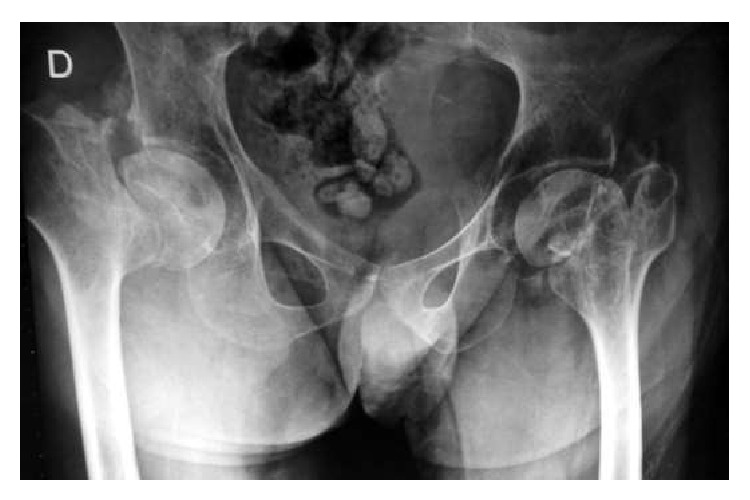
Anteroposterior both-hip radiograph showing bilateral Garden IV femoral neck fracture.

Anteroposterior and lateral hip X-rays were taken and a bilateral Garden 4 fracture was found [8] (Figure 1).

**Figure 2:**
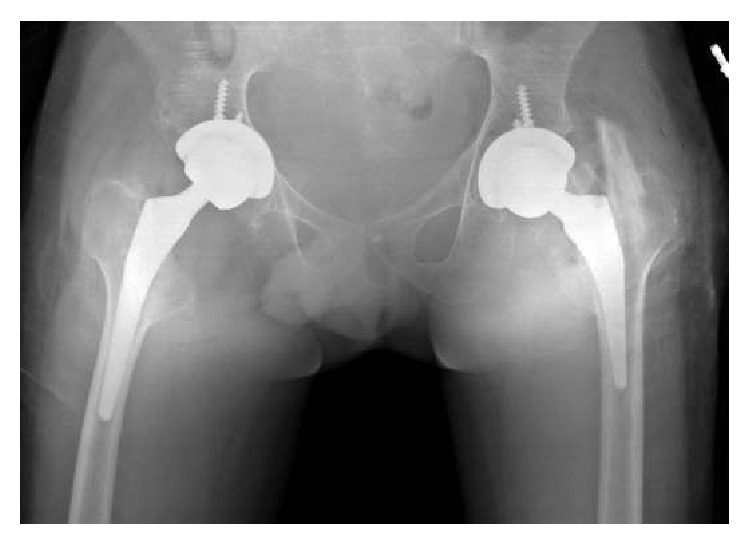
Anteroposterior both-hip radiograph depicting bilateral cemented THA at last follow-up.

Three days after admission, bilateral one-stage total hip arthroplasty under general anesthesia and through posterolateral approach was performed using cementless total hip prostheses (Trinity cup and Metafix stem, Corin, UK) with a 36 mm ceramic on polyethylene bearing surface (Figure 2).

After surgery, the patient was sent to intermediate care unit for pain management and sedation because of aggressive behavior.

During the second postoperative day he presented 38.5 degrees of axillary temperature and a* Proteus mirabilis* growth on peripheral blood cultures and he completed two weeks of intravenous ciprofloxacin covering a urinary tract infection. During the hospital stance the surgery wound showed no signs of infection. He was discharged after three weeks in intermediate care unit.

On 35th postoperative day the patient was seen in the outpatient clinic and surgical wound infection was observed. Irrigation and debridement under general anesthesia was performed. Tissue cultures showed growth of* Proteus mirabilis*,* Escherichia coli*, and a vancomycin-resistant* Enterococcus* (VRE). The patient was isolated from contact and initiated into an antibiotic scheme of linezolid and aztreonam during a six-week period without further complications.

After discharge he was referred to the endocrinology clinic and initiated into treatment with vitamin D supplements and zoledronic acid as an antiresorptive treatment. At last follow-up, 2 years after surgery, the patient was walking with his parents assistance and returned to his previous status, after he had completed an extensive rehabilitation program.

## 3. Discussion

Femoral neck fractures may occur when the bone is subjected to unusual loads or when a preexisting medical condition reduces bone strength [9], and the fact that it serves as a lever arm of the joint is a point of torque concentration [10].

Several reports emphasize bone disease as a major precipitating factor, and there is an increased incidence of hip fractures in chronic epilepsy [11], renal osteodystrophy, chronic steroid use, and transient osteopenia during pregnancy [3, 5, 11] (Table 1).

Our perspective in this patient is that the alterations of the calcium-phosphate metabolism could have precipitated the bilateral fracture, and although the decision making on the treatment is a matter of controversy, total hip arthroplasty (THA) preserves mobility and function for ambulatory patients with cerebral palsy [15, 16].

Femoral head resection has been proven to be effective in severely dislocated hip joints in completely immobilized patients [16], whereas this procedure was not a reasonable option in this patient who presented walking ability and moderate spasticity. Even in nonambulatory patients with cerebral palsy, Koffman reported on continuous postoperative pain in ten patients. Sitting tolerance was the only shown parameter to improve in that study [17].

Although THA offers the best option for pain relief and motion preservation, there have been concerns regarding the possibility of dislocation and component loosening, especially when performed in young adults or the mentally impaired persons [11, 15, 18, 19]. With advances in technique and prosthesis design, hip arthroplasty in patients younger than 50 years is becoming an accepted practice in the general population [14, 20, 21].

One concern may be the use of a cementless stem in this particular case. With recent improvements in uncemented fixation and developmental progress in the prosthesis structure, several groups have suggested the use of cementless femoral components in porotic bone. Kelly et al. [22] reported high clinical scores (median Harris hip score, 94.5) and no femoral fixation failures in 15 Class C (osteoporotic) bone patients with a minimum of a 9-year follow-up (average, 11.5 years; range, 9–14 years) and the use of a hydroxyapatite-coated cemented stem.

The treatment of enterococcal infections can be difficult and should involve a combination of agents that can synergistically confer bactericidal activity. Linezolid and daptomycin are reserved for serious VRE infections that are resistant to penicillins [23].

Despite the treatment election, no enough emphasis could be made on the importance of early diagnosis of this type of entities. Primary care physicians should be aware of and alert to the possibility of fractures in patients with neurological disorders and calcium metabolism alterations.

This population rapidly develops fixed flexion contractures when subjected to long periods of bed rest, which makes the rehabilitation process difficult. This fact enhances the necessity of an expeditious diagnosis of any osseous lesion. Late diagnosis of orthopedic injuries in this type of patients is a matter of great concern as it may lead to permanent disability [20].

In those cases surgery should help patients to rapidly regain mobilization and resume previous levels of independence, and the preservation of hip motion should permit better patient care.

Although infrequent, femoral neck fractures should be considered in cerebral palsy patients mimicking an acute abdominal episode. Total hip arthroplasty should be taken into consideration as a valid alternative in previously ambulatory cerebral palsy patients.

## Figures and Tables

**Table 1:** Reported cases of spontaneous femoral fractures.

Authors	Study level	Number of patients	Age/sex of the patient	Etiology	Comorbidities	Bilateral
Çopuroğlu et al. [12].	V	1	82 F	Epileptic seizure	Arrhythmia Epilepsy	Yes

Siddique et al. [13].	V	1	83 F	Osteopenia	Diabetes mellitus	Yes

Schnadower et al. [14].	V	1	17 M	Hypocalcemic convulsion	Primary vitamin D deficiency	Yes

Csotye^1^ et al. [3].	V	1	33 F	Transient osteoporosis in pregnancy	No	Yes

Siddique et al. [13].	V	1	30 F	Hypocalcemic convulsions	Chronic renal failure Secondary hyperparathyroidism	Yes

Ohishi et al. [5].	V	1	39 F	—	Chronic vitamin D deficiency	No

F: female. M: Male. ^1^Department of Traumatology, Bekes County Hospital, Gyula, Hungary.
